# How culture orientation influences the COVID-19 pandemic: An empirical analysis

**DOI:** 10.3389/fpsyg.2022.899730

**Published:** 2022-09-29

**Authors:** Zhuo Wang, Yi Li, Ruiqing Xu, Haoting Yang

**Affiliations:** ^1^Department of International Economics and Trade, School of Economics, Beijing Technology and Business University, Beijing, China; ^2^Stuart School of Business, Illinois Institute of Technology, Chicago, IL, United States; ^3^Department of Environmental Design, School of Art Design, Beijing University of Technology, Beijing, China

**Keywords:** collectivism-individualism, the COVID-19 pandemic, culture-related factors, policy responses, action pathway

## Abstract

**Purpose:**

This study aims to investigate the mediational path of the influence of cultural orientation on the COVID-19 pandemic outcome at the national level and find out whether some culture-related factors can have a moderating effect on the influence of culture.

**Methodology:**

Cultural dimension theory of Hofstede is used to quantify the degree of each dimension of culture orientation. The cross-section regression model is adopted to test if culture orientations affect the pandemic outcome, controlling for democracy, economy, education, population, age, and time. Then, a mediational analysis is conducted to examine if policy response is the mediator that culture makes an impact on the pandemic outcome. Finally, a moderation analysis is carried out to determine how each control variable has moderated the influence.

**Findings:**

The cross-section regression results showed that culture orientation influences the outcome of the COVID-19 pandemic at the 99% confidence level and that among the six cultural dimensions, collectivism-individualism has the most significant impact. It has also been found that policy response is the mediator of cultural influence, and culture-related factors can moderate the influence.

**Contribution:**

The contribution of this research lies in developing the assertion that culture influences pandemic outcomes. Our findings indicate that collectivism-individualism culture orientation affects the effectiveness of epidemic controls the most among the six culture dimensions. Additionally, our research is the first to study the mediating effect of policy responses and the moderating effect of culture-related factors on the influence of cultural orientation on the pandemic outcome.

## Introduction

The COVID-19 crisis has been a significant shock to the global economy and the livelihoods of citizens. Governments differ in responding to the pandemic and sustaining economic and social activities. Like the UK and the Netherlands, no significant measures have been taken to combat the spread of the virus or any tactics that seemed insufficient and ultimately proved ineffective (Xiao, 2021). However, in other countries, such as China and South Korea, public health officials have the power to enforce quarantines that have led to a dramatic drop in new infections. So far, both achievements and consequences of these measures have come into effect. Different ways of containing, suppressing, and mitigating adverse effects achieved quite different results.

There exist various factors that may contribute to the outcome of the pandemic. Researchers of different academic backgrounds, such as psychology, epidemiology, sociology, and so on, have attributed different causes to the pandemic. Among the various factors that may affect the pandemic outcome of a country, does culture play an essential role? If so, are different culture dimensions proposed by Hofstede stimulative or inhibitory? How do they act on the outcome of the response to the pandemic? Is this influence strengthened or weakened by other culture-related factors? These are the questions that this study seeks to address.

## Literature review

Current research on these significant regional differences is mainly based on epidemiology and government policy, with the former devoted to analyzing the epidemiological characteristics of the virus and the latter focusing on the political decisions during the pandemic. As another binding site of the prevention and control of the pandemic, the role of deep-seated cultural and psychological mechanisms played in the pandemic is less studied.

Cao et al. (2020) showed that the effectiveness of policies depends largely on other factors such as culture. However, the number of countries in their sample is only 55, and they are limited to the two dimensions of tightness-looseness and individualism-collectivism. Bok et al. (2021) found that high collectivism and global personal impact are associated with lower COVID-19 public policy hypocrisy. However, the COVID-19 public health hypocrisy can change over time. New variants and new cultural norms could alter cooperation in public health mitigation.

Different measures in the face of the epidemic, such as social distancing, mask use, self-quarantine, city lockdown, and so on, are directly related to collectivism and individualism culture differences. From the perspective of the individualism-collectivism dimension, Xiao (2021) argues that the reason for the effective control of the pandemic in East Asia is higher collectivism, which can also be considered civic responsibility (including a heightened concern for the health of others rather than individual freedom and convenience). Indeed, individualism-collectivism cultural dimension reflects the tradeoff one must make between self-interests and collective interests (Chen et al., 2021). However, individualism, which advocates the role of personal choice, personal freedom, and self-actualization, has been somewhat undermined during the pandemic lockdown.

In addition, Xiao (2021) suggested that a higher collectivism orientation may be associated with less psychological dysregulation in anxiety, stress, and emotional difficulties. However, Shekriladze et al. (2021) used a moderation analysis to show that both individualism and collectivism can reduce the effects of anxiety on coping during pandemics. People with high collectivism displayed significantly higher passive-submissive coping styles without anxiety. However, when anxiety was elevated, its facilitation of passive compliant coping increased in both conditions and was more pronounced in low collectivism conditions. But the main limitation of its study is its bias on convenience sampling, which limits the generalizability of the findings.

These results can be found in the behavior of people wearing masks at the beginning of the epidemic. Lu et al. (2021) found that more collectivist U.S. states had higher rates of mask use and greater compliance with state policies. But their study relied on self-reported mask use rather than personal observations of mask use, leading to potential bias in self-reported individual mask use.

Jiang et al. (2022) argue that as the pandemic develops, personal attitudes and government responses may change to suit the environment, while economic factors and technologies such as vaccines may play an increasingly important role. Thus, individualism vs. collectivism cultural distinctions can explain disparities in communication in the early stages of the pandemic. But they only take into account fundamental factors, while many specific factors are omitted.

In conclusion, many researchers have found a relationship between collectivism and COVID-19. Rajkumar (2021) argues that individualism is positively associated with COVID-19 morbidity, mortality, and case fatality. Maaravi et al. (2021) also found that the more individualistic a country was, the more COVID-19 cases and deaths it had. They found that the more individualistic participants were, the more likely they were not to comply with epidemic prevention measures. But there may be country-specific differences in the underlying mechanics, which provides a research direction for us.

In addition to collectivism, Duarte et al. (2022) argues that citizens in countries with greater power distance seem to be more receptive to hierarchical order, and specifically that people's respect for state authorities (including health professionals) and compliance with restrictive rules may lead to a higher level of commitment to restrictive rules and ultimately to a lower level of epidemic severity. Dzandu et al. (2022) found that less indulgent countries (e.g., France, Italy, and Germany) were more receptive to the app than countries such as the United Kingdom and the United States. In countries with low indulgence, people are more willing to curb self-gratification for their needs and adhere to strict social norms, such as government calls for COVID-19 contact-tracing apps to be used together to tackle the outbreak. However, there is a lack of data for emerging and developing countries to compare with data from selected developed countries. Chen and Biswas (2022) argue that countries with high uncertainty avoidance (e.g., France, Italy, Spain, Colombia, and Turkey) have been slow to implement control measures to stop the spread of the virus. Likewise, highly masculine cultures that do not properly adhere to control measures (e.g., Italy, India, Germany, and the United States) are associated with more confirmed cases and deaths. Sekar et al. (2022) also argue that the national cultural dimension explains about 66% of the variance in the initiative and found that low power distance, low masculinity, and high uncertainty avoidance were associated with the increased initiative against the pandemic. Lucas et al. (2022) found that cultural values were associated with support for vaccination and quarantine prevention policies, and they observed a relationship between beliefs in justice and national values (i.e., power distance, individualism, masculinity, uncertainty avoidance, long-term orientation, and indulgence), illustrating that the impact of belief in justice on mandates supporting COVID-19 behavior may depend on national context. For example, in countries with high levels of uncertainty avoidance, people will naturally avoid social gatherings, suggesting that coercive policies may be unnecessary. Since their within-country sample size is small, their study cannot be generalized to other countries.

Therefore, it is not enough to conclude that collectivism has a positive effect on epidemic prevention and control. Cultural explanations for the success of anti-epidemic measures in collective societies should not obscure the fact that effective governments in some individualistic countries have also achieved positive results in fighting the pandemic. A good example is the relative success of the Australian government in containing the pandemic compared to the US.

In March 2020, to control the surge in infections, Australia issued a ban, announcing that it would close its borders to everyone except its residents, and its citizens would not leave the country until they were immune. The draconian containment measures have earned the country the nickname “Australia's Fortress” (Stobart and Duckett, 2022). As a country with a high index of individualism, Australia ranks behind many individualist countries and not even higher than many collectivist countries in the number of confirmed cases. To explain this phenomenon, we need to consider the role of other culture-related factors in strengthening or weakening the influence of collectivist-Individualism culture.

While government measures may help curb the rise in cases, they do not provide the whole picture. The spread of infectious diseases in a society may also be related to its culture, which determines individual behavior and decision-making. Dheer et al. (2021) showed how case growth differed during the first wave of the pandemic in culturally diverse countries. It also highlights the moderating role of culture by illustrating the effect of culture on the growth rate of COVID-19 in countries with comparable levels of government rigor over time. Differences in reporting practices across countries still present limitations to the study, although they performed exponential smoothing to address issues such as discontinuities in daily case reporting and examined alternating 7-day moving averages.

Additionally, recent research has highlighted the impact of culture on social engagement in social distancing and self-isolation practices. However, their understanding of the role of culture in regulating the growth rate of COVID-19 across countries is incomplete. Developing this knowledge is necessary to unlock the deep-seated constraints that can hinder countries' fight against infectious diseases. From a policy perspective, this could allow governments to develop strategies that are not only in line with science but also understand how human behavior is influenced by cultural context, thereby increasing the likelihood that they will be successful in saving lives.

As of this writing, the pandemic is still ongoing, and the pattern of the pandemic has constantly been changing from the situation when it initially burst; for example, the novel coronavirus SARS-CoV-2 has mutated into a more infectious but less lethal variant—Omicron, therefore, a timely update of the sample is needed.

In this research report, we will combine insights from cross-cultural research with a survey of the social psychology and public health literature to advance theoretical underpinnings and culturally derived explanations of cross-country differences in COVID-19 outcomes. Specifically, we intend to argue that culture will affect the total confirmed and dead cases of COVID-19 by influencing society's beliefs about the legitimacy of mitigation measures.

A nation-specific culture often implies nation-specific ways of behaving. Previous studies generally recognize that the cultural influence on the pandemic outcome does exist (e.g., Cao et al., 2020; Bok et al., 2021; Dheer et al., 2021; Kumar, 2021; Lu et al., 2021; Rajkumar, 2021; Xiao, 2021; Chen and Biswas, 2022). Cultural factors are essential for public health officials to consider when initiating and maintaining adherence to public health measures (Lee et al., 2021). Psychologists have also posited that social norms and cultural characteristics influence human behavior for COVID-19 pandemic responses (Bavel et al., 2020).

The rest of the article is organized as follows: hypotheses, variable selection, data collection and methodology, analytic models and results, discussion, and conclusion.

## Hypotheses

In order to validate and develop the previous research findings, we propose the first hypothesis in this study:

**Hypothesis 1 (H1)**: Culture has a significant impact on the outcome of the pandemic.

Among all previous studies related to the cultural impact of the pandemic in the above literature review, the nation-level comparison of collectivism and individualism gets much attention. It leads us to the second hypothesis:

**Hypothesis 2 (H2)**: Collectivism-Individualism culture orientation has the most significant impact among all cultural dimensions on the outcome of the pandemic.

Culture and values are essential to public policies (Daniell, 2014; Muers, 2018). Meanwhile, governments make healthcare policy responses that make a difference in testing and attributing deaths (Greer et al., 2021). Since the culture of a country may influence the formulation of its policies, and the policies have a significant impact on the outcome of epidemic prevention and control, we conjecture the third hypothesis in this paper:

**Hypothesis 3 (H3)**: Policy response to the pandemic is a mediational factor of a Collectivism-Individualism culture that produces the impact.

As there are some counterexamples in reality, for example, some highly individualistic countries did well in the face of the pandemic, and this paper proposes the fourth hypothesis to explain these counterexamples:

**Hypothesis 4 (H4)**: Several culture-related variables have a moderation effect on the impact of Collectivism-Individualism culture orientation.

## Variable selection, data collection, and methodology

### Variable selection

Cultural difference is particularly evident in the performance of people worldwide in responding to the epidemic. Because fighting the virus requires the government to take measures, it also requires the cooperation of the people. The degree of cooperation of the people depends on their attitude toward the epidemic itself, the policies issued by the government, and whether they comply with various constraints on freedom when necessary. This degree is closely related to their cultural environment and background.

The culture dimension theory of Professor Geert Hofstede is one of the most comprehensive and recognized studies in the social psychology field. Initially, Hofstede only studied four dimensions: Individualism, Power Distance, Uncertainty Avoidance, and Masculinity. Research of posterity extended the dimensions by adding Long-Term Orientation and Indulgence. Hofstede published his research findings on the four dimensions in 1980, and the last two dimensions were, respectively, updated by Michael Bond and Micheal Minkov (Zhe, 2015). Currently, Hofstede's cultural dimension theory measures national cultural preferences in a specific country from six different dimensions, providing a benchmark for people to identify and understand cultural phenomena. At the same time, it provides an effective tool for comparative analysis of different cultures, enables researchers to grasp behavioral insights more quickly, and provides a foundation for cross-cultural management.

Although there are several studies against the cultural theory of Hofstede (Gerlach and Eriksson, 2021; Pelham et al., 2022) and many other cross-culture theories, more than half of the most recently published papers related to our topic used the data from Hofstede as a reference since 2021 (e.g., Chen and Biswas, 2022; Duarte et al., 2022; Dzandu et al., 2022; Jiang et al., 2022; Lucas et al., 2022; Sekar et al., 2022). At the same time, it is acknowledged in this study that there is no single truth in cross-cultural studies, but only that it is more useful (Zhe, 2015). Therefore, out of consideration for completeness and availability of data and uniformity of evaluation indicators, we also adopted Hofstede's cultural dimension theory to analyze what kind of cultural differences lead to different anti-epidemic effects from different angles.

The six cultural dimensions are used as independent variables in this study. According to the official definition of Hofstede Insights website, *Individualism* is defined as a preference for a loosely knit social framework in which individuals are expected to take care of only themselves and their immediate families. The high value of Individualism indicates that society tends to be more individualistic, and the low value indicates the opposite. *Power Distance* expresses the degree to which the less powerful members of a society accept and expect that power is distributed unequally. The Higher Power Distance index means people with lower status are more likely to follow orders from people with higher status, and lower means the opposite. *Uncertainty Avoidance* expresses the degree to which the members of a society feel uncomfortable with uncertainty and ambiguity. Countries with high Uncertainty Avoidance index hold rigid codes of belief and behavior, so they often need many rules and regulations to constrain uncertainty, and countries with a low index are more tolerant of ambiguity and uncertainty. The *Masculine* side of this dimension represents a preference in society for achievement, heroism, assertiveness, and material rewards for success. A society of high Masculinity is more competitive and challenging and a society of low Masculinity is more consensus-oriented and tender. *Long-Term Orientation* looks at how society considers respect for tradition and fulfilling social obligations. Societies that score low on this dimension prefer to maintain traditions and norms, and those with a culture that scores high, on the other hand, are more pragmatic and future-oriented. Finally, *Indulgence is* a society that allows relatively free gratification of basic and natural human drives related to enjoying life and having fun. A society with high Indulgence values the satisfaction of human needs and desires; in contrast, a society with low Indulgence sees the value in curbing one's desires and withholding pleasures to align more with societal norms. Scores of all dimensions range from 0 to 100.

In a fast-moving pandemic, determining which countries have made the most successful progress in fighting the outbreak is not a simple task. Comprehensively considering the control variables selected by previous studies (Dheer et al., 2021; Lu et al., 2021; Maaravi et al., 2021; Chen and Biswas, 2022) and out of the concern of the availability of data, this article selects six control variables that also impact the outcome of the pandemic mitigation to determine the impact of such cultural orientation. They are democracy, economy, education, population, age, and time, each represented by the Democracy Index, GDP per Capita, Literacy Rate, Population Density, Percentage of Population Aged above 65, and Time Difference from the First Case Being Confirmed in the world. Such selection not only includes the fundamental aspects of the society (democracy, economy, and education) but also takes the factors that may hugely affect the outcome of the pandemic (population, age, and time) into account.

This research also studies moderating effect of these selected control variables. The moderating effect is reflected in the influence of culture-related factors on the impact of culture on the outcome of the outbreak. Considering that each control variable is related to culture and has its own impact on the pandemic, they are also used as moderators in the moderation analysis.

When testing the mediating effect of policy responses on the pandemic outcome, we used the Stringency Intensity as the mediator, calculated by dividing the total number of cases by the Stringency Index. The Stringency Index is a composite measure based on nine response indicators, including school closures, workplace closures, and travel bans, rescaled to a value from 0 to 100 (100 = strictest), which can reflect the strictness of the policies a country takes in the face of the pandemic. However, only using the Stringency Index can cause an endogenous problem, that is, the more severe the epidemic, the more stringent the policy responses. Therefore, we introduced Stringency Intensity based on this index.

As for the dependent variable, which represents the outcome of each country, we selected Total Cases and Total Deaths. To reduce the absolute value of the data for easy calculation, we take the logarithm of the Total Cases and Total Death. In addition, we add 1 to the actual value at first to avoid the case where the logarithm does not exist when the value is 0.

### Data collection

“Our World in Data” tracks the impact of the pandemic for a comprehensive assessment. It has built country profiles for 207 countries to delve into the statistics of the COVID-19 pandemic in the world. Each profile includes interactive visualizations, explanations of the metrics presented, and details on the data source. The country profiles are updated daily, allowing scholars to explore the statistics of the COVID-19 pandemic in every country in the world. Therefore, we retrieved all variables except for the score of each culture dimension, Democracy Index, and Time Difference from the First Cases Being Confirmed from the “Our World in Data COVID-19” (OWDC) dataset. These indicators are daily recorded and spanned from March 2020 to April 2022.

Data for the scores of each dimension are retrieved from https://www.hofstede-insights.com/, where both the original study of Hofstede and posterity's extensive research findings are recorded. Democracy Index comes from The Economist Intelligence Unit's index of democracy: https://www.eiu.com/n/webinars/democracy-index-2021, one of the most comprehensive and commonly used indexes for measuring democracy degree. The Time of First Case Being Confirmed in each country was retrieved from https://github.com/owid/covid-19-data/tree/master/public/data, and the Time Difference is worked out by the authors by calculating the date difference between the first confirmed case in that country and the first confirmed case in the world on 31 December 2019.

Our sample includes 91 countries with data sources for all variables. To examine whether there is multicollinearity within the variables selected, a VIF test was conducted. The VIF of all variables is below the cutoff of 10, indicating the collinearity between variables is low. In particular, most of the variables are below five except for the percentage of the population aged above 65. Therefore, the selection of these variables does not lead to the problem of unstable regression coefficients.

After the collection and processing of data, the descriptive statistics and VIF value of all independent variables are shown in Table 1. We've listed all tables in the Supplementary material.

**Table 1:** Descriptive statistics and VIF value of all independent variables.

**Variables**	**(1)**	**(2)**	**(3)**	**(4)**	**(5)**	**(6)**
	***N***	**Mean**	**SD**	**Min**	**Max**	**VIF**
Power distance	70,770	64.50	20.60	11	100	3.12
Individualism	70,770	40.15	22.32	10	91	3.24
Masculinity	70,770	48.16	18.41	5	100	1.10
Uncertainty avoidance	70,770	68.06	21.12	8	100	2.18
Long term orientation	70,770	45.94	24.14	4	100	2.32
Indulgence	70,770	44.78	22.59	0	100	1.91
Stringency intensity	67,305	0.125	1.266	0	90.74	1.00
Democracy Index	70,770	6.282	2.078	1.950	9.750	2.85
Population density	69,958	316.6	1,123	3.202	7,916	2.02
Aged 65 older	69,958	12.32	6.299	2.405	27.05	5.25
GDP per capita	69,958	25,286	18,614	1,136	94,278	3.24
Literacy rate	70,770	91.58	13.26	28.70	100	2.11
Time difference	70,770	53.48	18.86	0	84	1.55

### Methodology

To validate Hypothesis 1, we used a cross-section regression model, with the culture dimensions being the independent variable, the number of confirmed cases and deaths being the dependent variable, and the Democracy Index, GDP per Capita, Literacy Rate, Population Density, Aged 65 Older, and Time Difference being the control variables. In addition, we add the control variables one by one to avoid the problem of endogeneity. To validate Hypothesis 2, we observed the coefficients of each culture dimension to see if the Individualism dimension has the most significant impact on the pandemic outcome. To validate Hypothesis 3, mediational analysis is applied, with the culture dimension being the independent variable, the Stringency Index being the dependent variable, and the Democracy Index, GDP per Capita, Literacy Rate, Population Density, Aged 65 Older, and Time Difference being the control variables. If the result is significant, it can be inferred that cultural orientation influences the pandemic outcome by influencing Policy Responses. To validate Hypothesis 4, moderation analysis is adopted, with the culture dimensions being the independent variable, the number of confirmed cases and deaths being the dependent variable, and the Democracy Index, GDP per Capita, Literacy Rate, Population Density, Aged 65 Older, and Time Difference being the moderating variables. If the result is significant, a positive coefficient indicates a strengthening effect and vice versa.

To demonstrate Hypotheses 1 and 2, one estimates the following regression model:


(1) $$\begin{array}{l} Y=\alpha_{1}+aCul+\chi_{1}DE+\delta_{1}ECO+\kappa_{1}EDU\;+\sigma_{1}POP\\ \;+\xi_{1}AGE+\omega_{1}TIME+\varepsilon_{1} \end{array}$$

where *Y* is the pandemic outcome represented by total cases and total deaths, α is the constant term, Cul is the six culture dimensions measured by the value of each score, DE is the democratic degree measured by Democracy Index, ECO is the economic environment measured by GDP per Capita, EDU is education measured by Literacy Rate, POP is population status measured by Population Density, AGE is the percentage of population aged above 65, Time Difference is the time difference between the first case being confirmed in that country and the first case discovered in the world, and ε is the estimated error.

Hypothesis 3 exhibits a mediating effect of policy responses. We applied the statistical mediation analysis approach as outlined by Hayes (2009) to demonstrate the mediator:


(2) $$\begin{array}{l} M=\alpha_{2}+bCul+\chi_{2}DE+\delta_{2}ECO+\kappa_{2}EDU+\sigma_{2}POP\\ \;+\xi_{2}AGE+\omega_{2}TIME+\varepsilon_{2} \end{array}$$

(3) $$\begin{array}{l} Y=\alpha_{3}+a^{\prime }Cul+cM+\chi_{3}DE+\delta_{3}ECO+\kappa_{3}EDU+\sigma_{3}POP\\ \;+\xi_{3}AGE+\omega_{3}TIME+\varepsilon_{3} \end{array}$$

where *M* is policy responses represented by Stringency Intensity. As can be inferred, among Equations (1–3), *a* quantifies the *total effect* of culture, *a*′ quantifies the *direct effect*, and b^*^c quantifies the *indirect effect* of culture on the pandemic outcome through policy responses.

Hypothesis 4 aims to determine the moderation effect of culture-related factors on the influence of cultural orientation. Interaction terms are included in the model for validation.


(4) $$\begin{array}{l} Y=\alpha_{4}+a^{\prime \prime }Cul+\chi_{4}DE+\delta_{4}ECO+\kappa_{4}EDU+\sigma_{4}POP\\ \;+\xi_{4}AGE+\omega_{4}TIME+\varphi ID\;*\;DE+\mu ID\;*\;ECO\\ \;+\varsigma ID\;*\;EDU+\tau ID\;*\;POP+\upsilon ID\;*\;AGE+\vartheta I\;*\;TIME\\ \;+\varepsilon_{4} \end{array}$$

where ID is the degree of individualism measured by the individualism index. Equation (4) allows the impact of individualism to be moderated by control variables.

## Analytic models and results

### Validation of hypotheses 1 and 2

Equation (1) determines the overall effect of cultural orientation and the magnitude of the impact of each culture dimension. The regression output for the estimation of (1) is as follows.

Table 2 shows that all culture dimensions positively influence total cases at the 99% confidence level. Among them, the absolute value of Individualism is the largest, indicating that the impact of Individualism on total cases is the largest compared with other cultural dimensions.

**Table 2:** Impact of 6 culture dimensions on total cases.

	**(1)**	**(2)**	**(3)**	**(4)**	**(5)**	**(6)**
	**lnTC**	**lnTC**	**lnTC**	**lnTC**	**lnTC**	**lnTC**
Individualism	0.0406^***^	0.0356^***^	0.0349^***^	0.0324^***^	0.0373^***^	0.0343^***^
	(79.60)	(70.98)	(68.35)	(58.13)	(65.53)	(62.72)
Power distance	0.0254^***^	0.0225^***^	0.0218^***^	0.0227^***^	0.0190^***^	0.0132^***^
	(47.61)	(41.93)	(39.71)	(40.97)	(33.99)	(26.81)
Masculinity	0.0140^***^	0.0147^***^	0.0148^***^	0.0152^***^	0.0144^***^	0.00994^***^
	(42.43)	(44.80)	(44.87)	(45.57)	(43.24)	(34.08)
Uncertainty avoidance	0.0147^***^	0.0137^***^	0.0121^***^	0.0102^***^	0.0158^***^	0.0318^***^
	(40.60)	(36.08)	(30.87)	(23.05)	(33.00)	(68.59)
Long term orientation	0.000822^***^	0.00574^***^	0.00422^***^	0.00425^***^	0.00888^***^	0.00521^***^
	(2.59)	(16.95)	(12.02)	(12.13)	(23.61)	(13.36)
Indulgence	0.00284^***^	0.00518^***^	0.00424^***^	0.00328^***^	0.00137^***^	0.00291^***^
	(8.11)	(14.78)	(12.41)	(9.41)	(4.00)	(9.39)
Democracy Index	−0.0233^***^	0.0113^**^	0.00407	0.00652	0.0718^***^	0.103^***^
	(−4.72)	(2.27)	(0.79)	(1.27)	(12.34)	(18.89)
GDP per capita		−0.00000377^***^	−0.00000582^***^	−0.00000141^**^	−0.00000133^**^	−0.00000434^***^
		(−7.08)	(−10.87)	(−2.28)	(−2.03)	(−7.46)
Literacy rate			0.00855^***^	0.00795^***^	0.0158^***^	0.0119^***^
			(11.64)	(10.76)	(22.25)	(18.03)
Population density				−0.000104^***^	−0.0000469^***^	−0.0000706^***^
				(−12.01)	(−5.36)	(−8.11)
Aged 65 older					−0.0691^***^	−0.112^***^
					(−33.67)	(−53.81)
Time difference						−0.0427^***^
						(−94.59)
*N*	70,405	69,599	69,599	69,599	69,599	69,599
r2_a	0.656	0.660	0.661	0.662	0.666	0.710

t statistics in parentheses.

** p < 0.05,

*** p < 0.01.

lnTC = ln(total cases + 1).

Table 3 shows that all culture dimensions positively influence total deaths at the 99% confidence level. Among them, the absolute value of Individualism is still the largest, indicating that the impact of Individualism on total deaths is the largest compared with other cultural dimensions.

**Table 3:** Impact of 6 culture dimensions on total deaths.

	**(1)**	**(2)**	**(3)**	**(4)**	**(5)**	**(6)**
	**lnTD**	**lnTD**	**lnTD**	**lnTD**	**lnTD**	**lnTD**
Individualism	0.0444^***^	0.0438^***^	0.0430^***^	0.0375^***^	0.0420^***^	0.0384^***^
	(80.46)	(81.04)	(78.30)	(62.14)	(69.53)	(64.53)
Power distance	0.0237^***^	0.0173^***^	0.0166^***^	0.0185^***^	0.0151^***^	0.00826^***^
	(40.13)	(29.22)	(27.30)	(29.89)	(23.70)	(14.71)
Masculinity	0.0187^***^	0.0198^***^	0.0199^***^	0.0208^***^	0.0201^***^	0.0150^***^
	(48.23)	(51.17)	(51.22)	(52.80)	(51.24)	(42.47)
Uncertainty avoidance	0.0262^***^	0.0228^***^	0.0212^***^	0.0169^***^	0.0221^***^	0.0399^***^
	(63.19)	(55.62)	(49.20)	(34.28)	(42.04)	(79.07)
Long term orientation	−0.00342^***^	0.00649^***^	0.00491^***^	0.00496^***^	0.00922^***^	0.00513^***^
	(−9.93)	(17.37)	(12.58)	(12.90)	(22.62)	(12.12)
Indulgence	0.00203^***^	0.00766^***^	0.00665^***^	0.00451^***^	0.00272^***^	0.00433^***^
	(5.04)	(18.79)	(16.42)	(10.77)	(6.62)	(12.09)
Democracy Index	−0.0673^***^	−0.0144^***^	−0.0226^***^	−0.0171^***^	0.0437^***^	0.0766^***^
	(−12.52)	(−2.64)	(−4.03)	(−3.05)	(6.87)	(13.30)
GDP per capita		−0.0000233^***^	−0.0000255^***^	−0.0000158^***^	−0.0000157^***^	−0.0000193^***^
		(−45.08)	(−48.25)	(−25.05)	(−23.79)	(−32.86)
Literacy rate			0.00910^***^	0.00782^***^	0.0150^***^	0.0111^***^
			(11.94)	(10.16)	(19.26)	(15.32)
Population density				−0.000232^***^	−0.000179^***^	−0.000211^***^
				(−30.00)	(−22.26)	(−27.62)
Aged 65 older					−0.0638^***^	−0.113^***^
					(−27.63)	(−47.39)
Time difference						−0.0482^***^
						(−94.70)
*N*	68,366	67,585	67,585	67,585	67,585	67,585
r2_a	0.458	0.465	0.466	0.471	0.476	0.548

t statistics in parentheses.

*** p < 0.01.

lnTD = ln(total deaths + 1).

The results show that all culture dimensions positively impact the total cases and deaths at the 99% confidence level. In other words, the higher the score of each cultural dimension, the higher the total number of confirmed cases and deaths. Hypothesis 1 is validated. It can also be learned that among all cultural dimensions, Individualism has the most significant impact, as a change in the Individualism index causes the most significant change in the number of total cases and deaths. Hypothesis 2 is validated.

**Figure 1:**
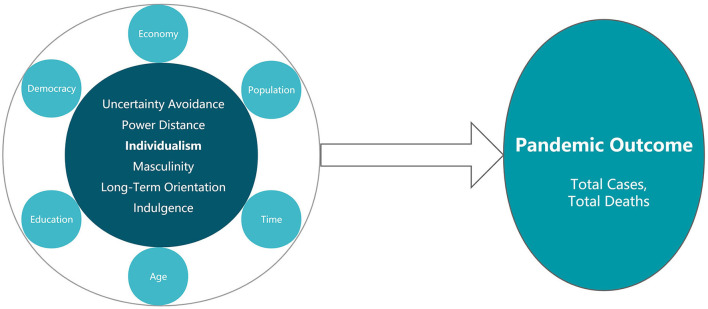
Mediating effect of policy response. Source: Drawn by the author.

The impact of the six culture dimensions and culture-related factors on the pandemic outcome can be expressed by the Figure 1 below:

### Validation of hypothesis 3

This model determines the mediating effect of policy responses, that is, the indirect effect of culture orientation *via* policy responses. The regression output for the estimation of Equation (2) is as follows.

It can be seen from Table 4 that Individualism has a positive impact on the policy response stringency at the 99% confidence level, which shows that the more individualistic a country, the more stringent are the policy responses. This may differ from our intuition, but since the influence of individualistic culture on policy stringency is not the focus of this article, it will not be explored in detail here.

**Table 4:** Impact of 6 culture dimensions on policy responses.

	**(1)**	**(2)**	**(3)**	**(4)**	**(5)**	**(6)**
	**Stringency intensity**	**Stringency intensity**	**Stringency intensity**	**Stringency intensity**	**Stringency intensity**	**Stringency intensity**
Individualism	0.000157	0.000614^**^	0.000648^**^	0.000937^***^	0.00128^***^	0.00156^***^
	(0.53)	(1.99)	(2.16)	(3.23)	(4.00)	(4.70)
Power distance	0.00112^***^	0.000707^***^	0.000732^***^	0.000627^**^	0.000365	0.00106^***^
	(4.28)	(2.64)	(2.71)	(2.26)	(1.29)	(3.62)
Masculinity	−0.00120^***^	−0.00114^***^	−0.00114^***^	−0.00119^***^	−0.00125^***^	−0.000760^***^
	(−6.77)	(−6.48)	(−6.48)	(−6.62)	(−6.95)	(−4.37)
Uncertainty avoidance	0.000587^***^	0.000306	0.000372^*^	0.000595^**^	0.000992^***^	−0.000706^**^
	(2.99)	(1.57)	(1.82)	(2.42)	(3.63)	(−2.35)
Long term orientation	−0.00105^***^	−0.000406^*^	−0.000343	−0.000347	−0.0000183	0.000421^*^
	(−5.92)	(−1.69)	(−1.24)	(−1.26)	(−0.07)	(1.71)
Indulgence	0.00100^***^	0.00141^***^	0.00145^***^	0.00156^***^	0.00143^***^	0.00123^***^
	(3.81)	(4.83)	(4.60)	(4.65)	(4.11)	(3.54)
Democracy Index	−0.0165^***^	−0.0139^***^	−0.0136^***^	−0.0139^***^	−0.00921^*^	−0.0117^**^
	(−3.78)	(−2.95)	(−2.82)	(−2.89)	(−1.95)	(−2.41)
GDP per capita		−0.00000233^***^	−0.00000224^***^	−0.00000275^***^	−0.00000276^***^	−0.00000246^***^
		(−7.67)	(−7.87)	(−7.74)	(−7.76)	(−6.82)
Literacy rate			−0.000373	−0.000305	0.000257	0.000728
			(−0.95)	(−0.79)	(0.57)	(1.55)
Population density				0.0000120^***^	0.0000161^***^	0.0000183^***^
				(2.79)	(3.61)	(4.10)
Aged 65 older					−0.00491^***^	−0.000319
					(−3.95)	(−0.26)
Time difference						0.00458^***^
						(12.63)
*N*	67,305	66,514	66,514	66,514	66,514	66,514
r2_a	0.162	0.163	0.163	0.163	0.163	0.166

t statistics in parentheses.

* p < 0.1,

** p < 0.05,

*** p < 0.01.

The regression output for the estimation of (3) is as follows.

Table 5 shows that the coefficient of the Stringency Index is obviously not close to 0 and its mediating effect between Individualism and Total Cases exists at the 99% confidence level, and it is, therefore, possible to infer that policy responses play a mediational role.

**Table 5:** Mediating effect of policy responses on total cases.

**Variables**	**(1)**	**(2)**	**(3)**
	**lnTC**	**Stringency intensity**	**lnTC**
Stringency intensity			−0.746^***^
			(0.00845)
Individualism	0.0336^***^	0.00185^***^	0.0342^***^
	(0.000897)	(0.000394)	(0.000858)
Power distance	0.0128^***^	0.00120^***^	0.0163^***^
	(0.000950)	(0.000421)	(0.000918)
Masculinity	0.0102^***^	−0.000866^***^	0.00948^***^
	(0.000625)	(0.000274)	(0.000598)
Uncertainty avoidance	0.0314^***^	−0.000617^*^	0.0308^***^
	(0.000774)	(0.000341)	(0.000743)
Long term orientation	0.00542^***^	0.000350	0.00695^***^
	(0.000714)	(0.000314)	(0.000685)
Indulgence	0.00291^***^	0.00125^***^	0.00352^***^
	(0.000679)	(0.000298)	(0.000650)
Democracy Index	0.0982^***^	−0.0109^***^	0.104^***^
	(0.00894)	(0.00394)	(0.00859)
GDP per capita	−4.40e−06^***^	−2.45e−06^***^	−6.80e−06^***^
	(1.07e−06)	(4.70e−07)	(1.02e−06)
Literacy rate	0.0120^***^	0.000631	0.0144^***^
	(0.00120)	(0.000528)	(0.00115)
Population density	−8.02e−05^***^	2.08e−05^***^	−7.35e−05^***^
	(1.39e−05)	(6.10e−06)	(1.33e−05)
Aged 65 older	−0.111^***^	−0.000620	−0.110^***^
	(0.00400)	(0.00176)	(0.00383)
Time difference	−0.0311^***^	0.00103^***^	−0.0288^***^
	(0.000732)	(0.000326)	(0.000710)
Constant	7.976^***^	0.00196	7.485^***^
	(0.135)	(0.0605)	(0.132)
Observations	69,599	66,514	66,514
*R*-squared	0.085	0.002	0.183

Standard errors in parentheses.

*** p < 0.01,

* p < 0.1.

lnTC = ln(total cases + 1).

As can be seen from Table 6, the same is true for Total Deaths. Stringency Intensity is the mediation between Individualism Index and Total Deaths. Moreover, the negative coefficient shows that the more extreme stringency, the fewer confirmed deaths.

**Table 6:** Mediating effects of policy responses on total deaths.

**Variables**	**(1)**	**(2)**	**(3)**
	**lnTD**	**Stringency intensity**	**lnTD**
Stringency intensity			−1.678^***^
			(0.0293)
Individualism	0.0386^***^	0.00185^***^	0.0383^***^
	(0.000759)	(0.000394)	(0.000750)
Power distance	0.00862^***^	0.00120^***^	0.0122^***^
	(0.000807)	(0.000421)	(0.000805)
Masculinity	0.0138^***^	−0.000866^***^	0.0135^***^
	(0.000528)	(0.000274)	(0.000522)
Uncertainty avoidance	0.0386^***^	−0.000617^*^	0.0374^***^
	(0.000658)	(0.000341)	(0.000652)
Long term orientation	0.00611^***^	0.000350	0.00696^***^
	(0.000603)	(0.000314)	(0.000598)
Indulgence	0.00567^***^	0.00125^***^	0.00541^***^
	(0.000574)	(0.000298)	(0.000568)
Democracy Index	0.0650^***^	−0.0109^***^	0.0810^***^
	(0.00757)	(0.00394)	(0.00752)
GDP per capita	−2.03e−05^***^	−2.45e−06^***^	−2.21e−05^***^
	(9.04e−07)	(4.70e−07)	(8.95e−07)
Literacy rate	0.0112^***^	0.000631	0.0142^***^
	(0.00101)	(0.000528)	(0.00101)
Population density	−0.000213^***^	2.08e−05^***^	−0.000214^***^
	(1.18e−05)	(6.10e−06)	(1.17e−05)
Aged 65 older	−0.111^***^	−0.000620	−0.112^***^
	(0.00338)	(0.00176)	(0.00335)
Time difference	−0.0440^***^	0.00103^***^	−0.0416^***^
	(0.000622)	(0.000326)	(0.000623)
Constant	4.928^***^	0.00196	4.394^***^
	(0.115)	(0.0605)	(0.115)
Observations	67,596	66,514	64,567
*R*-squared	0.189	0.002	0.232

Standard errors in parentheses.

*** p < 0.01,

* p < 0.1.

lnTD = ln(total deaths + 1).

What can be seen from this result is that no matter which result variable is used to represent the pandemic outcome, policy responses do have a mediating effect on them at the 99% confidence level. Also, it implies that the more stringent the policy responses, the fewer the confirmed cases and deaths. Hypothesis 3 is validated.

However, it is also worth noting that since the coefficient of Individualism did not turn into 0 in equation (3), the Stringency Intensity is not the perfect mediation between Individualism and pandemic outcome. Other mediational factors may lead Collectivism-Individualism culture to impact the pandemic mitigation. This article presents only one of these paths.

### Validation of hypothesis 4

Equation (4) is designed to examine the moderating effect of the six control variables selected in the beginning. This solves the concern that not all real-life examples abide by hypotheses 1 and 2. The regression output of Equation (4) is as follows:

Table 7 shows that Democracy Index, Literacy Rate, and Time Difference from the First Case Being Confirmed have a negative moderating effect on the influence of Collectivism-Individualism culture orientation on Total Cases and Total Deaths. In contrast, GDP per Capita and Percentage of the Population Aged above 65 have a weak positive moderating effect at the 99% confidence level. Population Density negatively moderates the influence on Total Cases at a confidence level <90%. However, it has a positive moderating effect at the 99% confidence level. Among these factors, Democracy Index has the most significant absolute value of the coefficient, so its moderating effect on democracy is the greatest among all control variables selected.

**Table 7:** Moderating effect of control variables on total cases.

**Variables**	**(1)**	**(2)**
	**lnTC**	**lnTD**
Individualism	0.293^***^	0.337^***^
	(0.00813)	(0.00681)
Democracy Index	0.625^***^	0.775^***^
	(0.0178)	(0.0149)
GDP per Capita	2.40e−06	−2.96e−05^***^
	(2.74e−06)	(2.31e−06)
Literacy Rate	0.0441^***^	0.0526^***^
	(0.00264)	(0.00221)
Population Density	−0.000244^***^	−0.000567^***^
	(4.88e−05)	(4.12e−05)
Aged 65 older	−0.0720^***^	−0.0956^***^
	(0.00671)	(0.00561)
Time Difference	0.0174^***^	0.0157^***^
	(0.00133)	(0.00113)
IDDE	−0.0207^***^	−0.0266^***^
	(0.000503)	(0.000422)
IDECO	1.95e−07^***^	6.61e−07^***^
	(5.51e−08)	(4.63e−08)
IDEDU	−0.000953^***^	−0.00109^***^
	(9.72e−05)	(8.15e−05)
IDPOP	−3.02e−06	5.94e−06^***^
	(1.87e−06)	(1.57e−06)
IDAGE	0.000852^***^	0.00170^***^
	(0.000166)	(0.000139)
IDTIME	−0.00112^***^	−0.00137^***^
	(3.08e−05)	(2.60e−05)
Constant	2.929^***^	−1.513^***^
	(0.232)	(0.194)
Observations	69,599	67,596
*R*-squared	0.093	0.211

t statistics in parentheses.

*** p < 0.01.

lnTC = ln(total cases + 1), lnTD = ln(total deaths + 1), IDDE = Individualism Index ^*^ Democracy Index, IDECO = Individualism Index^*^GDP per Capita. IDEDU = Individualism Index^*^Literacy Rate. IDPOP = Individualism Index^*^Population Density. IDAGE = Individualism Index^*^Percentage of Population Aged above 65. IDTIME = Individualism Index^*^Time Difference.

As can be seen from the results, democracy, education, and time negatively moderate the influence of collectivism-individualism culture orientation at the 99% confidence level. In contrast, the economy and age have a positive moderating effect at the same confidence level. This shows that even though collectivism-individualism culture orientation plays a vital role in mitigating the pandemic, it is also moderated by other culture-related factors. Among these factors, the degree of democracy of a nation has the most prominent effect. In addition, the pandemic mitigation outcome is determined by a comprehensive effect of all factors, not just simply relying on a single influential factor. In this way, Hypothesis 4 has been validated.

**Figure 2:**
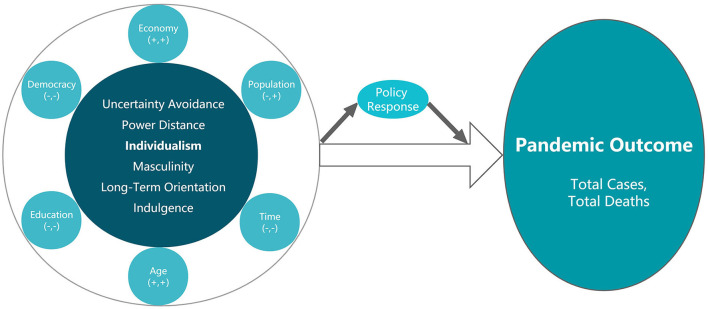
Moderating effect of each control variable. Source: Drawn by the author. “+” and “–” in parentheses, respectively, indicate positive or negative effects of the moderating effects on the influence of culture on Total Cases and Total Deaths. For example, the economy has a positive moderating effect on both, democracy has a negative moderating effect on both, and population has a negative moderating effect on the former but positive on the latter.

The moderating effect of each control variable can be expressed by the Figure 2 below:

### Robustness test

To test the stability of our research results, a robustness test has been conducted to prove the stability and reliability of our model.

This paper shortened the observation period to test the robustness of the model to prevent the contingency of the conclusion and enhance the model's reliability. The regression output from our model with all the daily data from 2021 onwards is as follows:

In Table 8, column (1) shows the regression result on the total cases. As it shows, the confidence level of all results has remained at 99%, and the coefficient sign of each culture dimension also remains. Column (2) shows the regression result on the total deaths, where the confidence level of all results has also remained at 99%, and the coefficient sign of each culture dimension stays positive. Apart from this, the coefficient of Individualism remained the largest, indicating that Individualism is still the cultural factor that has the most significant influence on pandemic performance. Column (3) shows the regression result on Stringency Intensity, where the confidence level of all results has remained at the 99%, and the sign of the coefficients of Masculinity, Uncertainty Avoidance, Long-Term Orientation, and Indulgence stay positive. However, Individualism and Masculinity both have changed the coefficient sign to negative. Since the impact of cultural factors on Policy Responses is not the core study of our research, such change does not affect the overall robustness of our model.

**Table 8:** Impact of 6 culture dimensions on total cases, total deaths, and the mediator.

	**(1)**	**(2)**	**(3)**
	**lnTC**	**lnTD**	**Stringency intensity**
Individualism	0.0370^***^	0.0379^***^	−0.00000943^***^
	(57.00)	(54.20)	(−10.23)
Power distance	0.0184^***^	0.0134^***^	−0.0000321^***^
	(32.45)	(20.90)	(−30.02)
Masculinity	0.0104^***^	0.0179^***^	−0.00000381^***^
	(31.57)	(44.70)	(−8.52)
Uncertainty avoidance	0.0279^***^	0.0376^***^	−0.0000241^***^
	(54.28)	(68.63)	(−21.98)
Long term orientation	0.00230^***^	0.00249^***^	0.00000600^***^
	(5.21)	(5.26)	(10.28)
Indulgence	0.00136^***^	0.000883^**^	0.00000346^***^
	(3.87)	(2.20)	(10.16)
Democracy Index	0.118^***^	0.113^***^	−0.0000777^***^
	(21.71)	(19.03)	(−8.53)
GDP per capita	−0.0000138^***^	−0.0000294^***^	−2.68e−08^***^
	(−21.37)	(−45.22)	(−21.24)
Literacy rate	0.0202^***^	0.0235^***^	0.0000139^***^
	(26.08)	(27.64)	(10.72)
Population density	−0.000119^***^	−0.000182^***^	0.000000299^***^
	(−11.96)	(−20.93)	(20.94)
Aged 65 older	−0.0877^***^	−0.100^***^	−0.0000184^***^
	(−39.16)	(−38.57)	(−7.73)
Time difference	−0.0352^***^	−0.0392^***^	0.00000510^***^
	(−69.90)	(−68.83)	(5.37)
*N*	41,490	41,490	39,311
r2_a	0.364	0.373	0.0962

t statistics in parentheses.

** p < 0.05,

*** p < 0.01.

lnTC = ln(total cases + 1), lnTD = ln(total deaths + 1).

In Table 9, column (1) shows the regression result on the total cases, the confidence level of all results has remained at the 99%, and the sign of the coefficients of Stringency Intensity and Individualism Index are the same. Column (2) shows the regression result on the total deaths, where the confidence level of all results has also remained at 99%, and the coefficient sign of the Stringency Intensity and Individualism Index stays the same.

**Table 9:** Mediating effect of stringency intensity.

**Variables**	**(1)**	**(2)**
	**lnTC**	**lnTD**
Stringency intensity	−344.1^**^	−334.0^**^
	(2.574)	(2.867)
Individualism	0.0328^**^	0.0340^**^
	(0.000545)	(0.000607)
Power distance	0.0102^**^	0.00527^**^
	(0.000579)	(0.000645)
Masculinity	0.00909^**^	0.0167^**^
	(0.000376)	(0.000419)
Uncertainty avoidance	0.0192^**^	0.0291^**^
	(0.000470)	(0.000523)
Long term orientation	0.00606^**^	0.00618^**^
	(0.000430)	(0.000479)
Indulgence	0.00236^**^	0.00160^**^
	(0.000407)	(0.000453)
Democracy Index	0.101^**^	0.0966^**^
	(0.00517)	(0.00576)
GDP per capita	−2.41e−05^**^	−3.94e−05^**^
	(6.47e−07)	(7.20e−07)
Literacy rate	0.0277^**^	0.0311^**^
	(0.000725)	(0.000808)
Population density	−2.93e−05^**^	−9.53e−05^**^
	(8.47e−06)	(9.43e−06)
Aged 65 older	−0.0929^**^	−0.105^**^
	(0.00236)	(0.00262)
Time difference	−0.0318^**^	−0.0355^**^
	(0.000450)	(0.000501)
Constant	9.638^**^	5.226^**^
	(0.0832)	(0.0927)
Observations	39,311	39,311
*R*-squared	0.511	0.514

Standard errors in parentheses.

*** p < 0.01.

Table 10 shows the regression results on the total cases. The confidence level of all results has remained at 99% except for GDP per Capita, which is at a 95% level. The results are generally in keeping with the previous result since GDP per Capita also showed a low confidence level in the previous moderation analysis. It is worth noting that the signs of the coefficients of all interaction terms are the same as before. Only the interaction term of Population Density did not reach the 90% confidence level. This is also in keeping with the significant result in the previous moderation analysis. Column (2) shows the regression result on the total deaths, where coefficient signs and the confidence level of all results have stayed the same as before. Among the interaction terms, the moderating effect of the Democracy Index is still the largest since it has the most significant coefficient value.

**Table 10:** Moderating effect of control variables.

**Variables**	**(1)**	**(2)**
	**lnTC**	**lnTD**
Individualism	0.329^***^	0.362^***^
	(0.00573)	(0.00613)
Democracy Index	0.746^***^	0.905^***^
	(0.0126)	(0.0134)
GDP per capita	−4.51e−06**	−3.01e−05^***^
	(1.92e−06)	(2.05e−06)
Literacy rate	0.0545^***^	0.0602^***^
	(0.00184)	(0.00197)
Population density	−0.000369^***^	−0.000763^***^
	(3.37e−05)	(3.61e−05)
Aged 65 older	−0.0811^***^	−0.0895^***^
	(0.00461)	(0.00494)
Time difference	0.0141^***^	0.0212^***^
	(0.000939)	(0.00100)
IDDE	−0.0241^***^	−0.0300^***^
	(0.000371)	(0.000397)
IDECO	2.03e−07^***^	5.26e−07^***^
	(3.92e−08)	(4.20e−08)
IDEDU	−0.00109^***^	−0.00102^***^
	(6.76e−05)	(7.24e−05)
IDPOP	1.23e−06	1.47e−05^***^
	(1.29e−06)	(1.38e−06)
IDAGE	0.00135^***^	0.00163^***^
	(0.000114)	(0.000122)
IDTIME	−0.00117^***^	−0.00143^***^
	(2.17e−05)	(2.32e−05)
Constant	3.015^***^	−2.278^***^
	(0.163)	(0.174)
Observations	41,490	41,490
*R*-squared	0.320	0.369

t statistics in parentheses.

*** p < 0.01.

lnTC = ln(Total Cases + 1), lnTD = ln(Total Deaths + 1), IDDE = Individualism Index ^*^ Democracy Index, IDECO = Individualism Index^*^GDP per Capita. IDEDU = Individualism Index^*^Literacy Rate. IDPOP = Individualism Index^*^Population Density. IDAGE = Individualism Index^*^Percentage of Population Aged above 65. IDTIME = Individualism Index^*^Time Difference.

In addition, the authors have also conducted robustness tests on samples in the observation period of the past 6 and 3 months, respectively. The results show that the value of the independent variable coefficient would change within a normal range, but the sign and confidence level have generally stayed the same as before. The selection of the sample observation period would affect the exact value of the results but does not affect the core conclusion of this study. Due to space limitations, the robustness test results of the observation period of these two are omitted here but can be obtained from the author if necessary.

In summary, all results have passed the robustness tests, which further proved the stability and reliability of the model.

## Discussion and conclusion

First, based on the cultural dimension theory of Hofstede, this paper validated that cultural orientation impacts the outcome of pandemics. We examined the relationship between cultural orientation and Total Cases and Deaths under a cross-section regression model while controlling for six other culture-related factors, such as democracy, economy, education, population density, age, and time. The result shows that all six dimensions significantly impact the number of confirmed cases and deaths.

Second, this article determined the factor with the most significant influence by comparing each dimension's coefficients. Individualism has the most significant coefficient, implying that a unit's change in Individualism caused the most remarkable change in the outcome of pandemic performance.

Third, this article examined the mediating effect of policy responses using the mediational analysis. The results show that part of the overall impact of Collectivism-Individualism culture orientation on the pandemic outcome is made *via* policy responses. In contrast, the culture itself directly makes the residual effect.

Finally, given that some counterexamples do not conform to Hypotheses 1 and 2, this article also considered whether the six control variables of Hypothesis 1 could impact the influence of cultural orientation. In other words, we examined the moderating effect of culture-related factors. The result shows that democracy, education, and time are negatively moderated. That is to say that these three factors can weaken the effect that Collectivism-Individualism culture orientation has on pandemic outcomes. It is also found that the economy and age have a positive moderating effect, though the effect is slight. However, no matter whether the effect is significant or slight, the pandemic outcome is forced by the mixed effects of all these factors, not simply by one of them.

The article sheds light on why countries differ in the performance of pandemic mitigation. Based on the cultural dimension theory of Hofstede, this model can well explain the fact that some countries of one orientation generally performed better than those of another. Also, this model gives evidence of the mediating effect of policy responses, showing that part of the influence of cultural orientation is generated through policy responses. Finally, this model further proved that democracy, economy, education, population density, age, and time are all moderating factors of Collectivism-Individualism culture orientation. Also, the pandemic outcome is an overall outcome of various culture and culture-related factors.

The uniqueness of this study is that it not only studied the impact of culture on the pandemic outcome as a whole but also broke down the culture into six different dimensions and determined the dimension with the most significant impact. In addition, the study further developed previous research which has focused on similar topics by examining the mediating effect of public policies and the moderation effect of culture-related factors. This complements the previous study that only a country with high collectivism index can successfully control the pandemic.

However, it has some limitations. For one thing, there are many other social factors related to culture, which may have different degrees of moderating effects on the impact of culture on the outcome of the pandemic. Limited by space, this paper only selected six representative ones as control variables based on summarizing previous studies. More relevant factors can be added as control variables in future studies. For another, Hofstede's evaluation of each cultural dimension of various countries was collected in the 1980s in a minimal sample, so its accuracy and universality in today's society are questioned. However, Hofstede's findings represent a plausible starting point as we try to figure out cultural differences and how different cultures affect pandemics. To balance data availability and sample coverage, this study assumes that culture has not changed substantially over the past decades and therefore used Hofstede's national data. If future studies can realize the extension of the broad concept of culture and the reconstruction of cultural dimensions and establish a new evaluation system based on the existing international statistical data, a more comprehensive innovation in theories and methods can be achieved.

## Data availability statement

The original contributions presented in the study are included in the article/Supplementary material, further inquiries can be directed to the corresponding authors.

## Author contributions

All authors listed have made a substantial, direct, and intellectual contribution to the work and approved it for publication.

## Conflict of interest

The authors declare that the research was conducted in the absence of any commercial or financial relationships that could be construed as a potential conflict of interest.

## Publisher's note

All claims expressed in this article are solely those of the authors and do not necessarily represent those of their affiliated organizations, or those of the publisher, the editors and the reviewers. Any product that may be evaluated in this article, or claim that may be made by its manufacturer, is not guaranteed or endorsed by the publisher.
