# Supplementary material for: How culture orientation influences the COVID-19 pandemic: An empirical analysis
Source: Front Psychol. 2022 Sep 29;13:899730. doi: 10.3389/fpsyg.2022.899730 (PMC9559590; doi:10.3389/fpsyg.2022.899730)
Supplement: Supplementary file 2 [file Data_Sheet_2.docx]

ssc install reghdfe

gen lnTC=ln(total_cases+1)

gen lnTD=ln(total_deaths+1)

regress lnTC individualism powerdistance masculinity uncertaintyavoidance longtermorientation indulgence democracyindex2021 gdp_per_capita literacyrateciafactbook2016 population_density aged_65_older time

estat vif

regress lnTD individualism powerdistance masculinity uncertaintyavoidance longtermorientation indulgence democracyindex2021 gdp_per_capita literacyrateciafactbook2016 population_density aged_65_older time

estat vif

*First step

reghdfe lnTC individualism powerdistance masculinity uncertaintyavoidance longtermorientation indulgence democracyindex2021,absorb(date) vce(r)

est sto WRW1

reghdfe lnTC individualism powerdistance masculinity uncertaintyavoidance longtermorientation indulgence democracyindex2021 gdp_per_capita,absorb(date) vce(r)

est sto WRW2

reghdfe lnTC individualism powerdistance masculinity uncertaintyavoidance longtermorientation indulgence democracyindex2021 gdp_per_capita literacyrateciafactbook2016,absorb(date) vce(r)

est sto WRW3

reghdfe lnTC individualism powerdistance masculinity uncertaintyavoidance longtermorientation indulgence democracyindex2021 gdp_per_capita literacyrateciafactbook2016 population_density,absorb(date) vce(r)

est sto WRW4

reghdfe lnTC individualism powerdistance masculinity uncertaintyavoidance longtermorientation indulgence democracyindex2021 gdp_per_capita literacyrateciafactbook2016 population_density aged_65_older,absorb(date) vce(r)

est sto WRW5

reghdfe lnTC individualism powerdistance masculinity uncertaintyavoidance longtermorientation indulgence democracyindex2021 gdp_per_capita literacyrateciafactbook2016 population_density aged_65_older time,absorb(date) vce(r)

est sto WRW6

esttab WRW1 WRW2 WRW3 WRW4 WRW5 WRW6 using Panel1.rtf, ///

starlevels(* 0.1 ** 0.05 *** 0.01 ) scalars() s(N r2_a ) ///

o(individualism powerdistance masculinity uncertaintyavoidance longtermorientation indulgence democracyindex2021 gdp_per_capita literacyrateciafactbook2016 population_density aged_65_older time) ///

k(individualism powerdistance masculinity uncertaintyavoidance longtermorientation indulgence democracyindex2021 gdp_per_capita literacyrateciafactbook2016 population_density aged_65_older time) ///

nogaps compress replace

reghdfe lnTD individualism powerdistance masculinity uncertaintyavoidance longtermorientation indulgence democracyindex2021,absorb(date) vce(r)

est sto WRW7

reghdfe lnTD individualism powerdistance masculinity uncertaintyavoidance longtermorientation indulgence democracyindex2021 gdp_per_capita,absorb(date) vce(r)

est sto WRW8

reghdfe lnTD individualism powerdistance masculinity uncertaintyavoidance longtermorientation indulgence democracyindex2021 gdp_per_capita literacyrateciafactbook2016,absorb(date) vce(r)

est sto WRW9

reghdfe lnTD individualism powerdistance masculinity uncertaintyavoidance longtermorientation indulgence democracyindex2021 gdp_per_capita literacyrateciafactbook2016 population_density,absorb(date) vce(r)

est sto WRW10

reghdfe lnTD individualism powerdistance masculinity uncertaintyavoidance longtermorientation indulgence democracyindex2021 gdp_per_capita literacyrateciafactbook2016 population_density aged_65_older,absorb(date) vce(r)

est sto WRW11

reghdfe lnTD individualism powerdistance masculinity uncertaintyavoidance longtermorientation indulgence democracyindex2021 gdp_per_capita literacyrateciafactbook2016 population_density aged_65_older time,absorb(date) vce(r)

est sto WRW12

esttab WRW7 WRW8 WRW9 WRW10 WRW11 WRW12 using Panel2.rtf, ///

starlevels(* 0.1 ** 0.05 *** 0.01 ) scalars() s(N r2_a ) ///

o(individualism powerdistance masculinity uncertaintyavoidance longtermorientation indulgence democracyindex2021 gdp_per_capita literacyrateciafactbook2016 population_density aged_65_older time) ///

k(individualism powerdistance masculinity uncertaintyavoidance longtermorientation indulgence democracyindex2021 gdp_per_capita literacyrateciafactbook2016 population_density aged_65_older time) ///

nogaps compress replace

*Second step

gen StringencyIntensity=stringency_index/total_cases

reghdfe StringencyIntensity individualism powerdistance masculinity uncertaintyavoidance longtermorientation indulgence democracyindex2021,absorb(date) vce(r) //1%显著

est sto WRW13

reghdfe StringencyIntensity individualism powerdistance masculinity uncertaintyavoidance longtermorientation indulgence democracyindex2021 gdp_per_capita,absorb(date) vce(r)

est sto WRW14

reghdfe StringencyIntensity individualism powerdistance masculinity uncertaintyavoidance longtermorientation indulgence democracyindex2021 gdp_per_capita literacyrateciafactbook2016,absorb(date) vce(r)

est sto WRW15

reghdfe StringencyIntensity individualism powerdistance masculinity uncertaintyavoidance longtermorientation indulgence democracyindex2021 gdp_per_capita literacyrateciafactbook2016 population_density,absorb(date) vce(r)

est sto WRW16

reghdfe StringencyIntensity individualism powerdistance masculinity uncertaintyavoidance longtermorientation indulgence democracyindex2021 gdp_per_capita literacyrateciafactbook2016 population_density aged_65_older,absorb(date) vce(r)

est sto WRW17

reghdfe StringencyIntensity individualism powerdistance masculinity uncertaintyavoidance longtermorientation indulgence democracyindex2021 gdp_per_capita literacyrateciafactbook2016 population_density aged_65_older time,absorb(date) vce(r)

est sto WRW18

esttab WRW13 WRW14 WRW15 WRW16 WRW17 WRW18 using Panel3.rtf, ///

starlevels(* 0.1 ** 0.05 *** 0.01 ) scalars() s(N r2_a ) ///

o(individualism powerdistance masculinity uncertaintyavoidance longtermorientation indulgence democracyindex2021 gdp_per_capita literacyrateciafactbook2016 population_density aged_65_older time) ///

k(individualism powerdistance masculinity uncertaintyavoidance longtermorientation indulgence democracyindex2021 gdp_per_capita literacyrateciafactbook2016 population_density aged_65_older time) ///

nogaps compress replace

*Third step

ssc inst outreg2

reghdfe lnTC StringencyIntensity individualism powerdistance masculinity uncertaintyavoidance longtermorientation indulgence democracyindex2021 gdp_per_capita literacyrateciafactbook2016 population_density aged_65_older time,absorb(date) vce(r)

quietly reg lnTC individualism powerdistance masculinity uncertaintyavoidance longtermorientation indulgence democracyindex2021 gdp_per_capita literacyrateciafactbook2016 population_density aged_65_older time

outreg2 using Mediate1,word replace

quietly reg StringencyIntensity individualism powerdistance masculinity uncertaintyavoidance longtermorientation indulgence democracyindex2021 gdp_per_capita literacyrateciafactbook2016 population_density aged_65_older time

outreg2 using Mediate1,word

quietly reg lnTC StringencyIntensity individualism powerdistance masculinity uncertaintyavoidance longtermorientation indulgence democracyindex2021 gdp_per_capita literacyrateciafactbook2016 population_density aged_65_older time

outreg2 using Mediate1,word

reghdfe lnTD StringencyIntensity individualism powerdistance masculinity uncertaintyavoidance longtermorientation indulgence democracyindex2021 gdp_per_capita literacyrateciafactbook2016 population_density aged_65_older time,absorb(date) vce(r)

quietly reg lnTD individualism powerdistance masculinity uncertaintyavoidance longtermorientation indulgence democracyindex2021 gdp_per_capita literacyrateciafactbook2016 population_density aged_65_older time

outreg2 using Mediate2,word replace

quietly reg StringencyIntensity individualism powerdistance masculinity uncertaintyavoidance longtermorientation indulgence democracyindex2021 gdp_per_capita literacyrateciafactbook2016 population_density aged_65_older time

outreg2 using Mediate2,word

quietly reg lnTD individualism powerdistance masculinity uncertaintyavoidance longtermorientation indulgence democracyindex2021 gdp_per_capita literacyrateciafactbook2016 population_density aged_65_older time

outreg2 using Mediate2,word

*Fourth step

gen IDDE=individualism*democracyindex2021

gen IDECO=individualism*gdp_per_capita

gen IDEDU=individualism*literacyrateciafactbook2016

gen IDPOP=individualism*population_density

gen IDAGE=individualism*aged_65_older

gen IDTIME=individualism*time

regress lnTC individualism democracyindex2021 gdp_per_capita literacyrateciafactbook2016 population_density aged_65_older time IDDE IDECO IDEDU IDPOP IDAGE IDTIME

outreg2 using Moderate1,word replace

regress lnTD individualism democracyindex2021 gdp_per_capita literacyrateciafactbook2016 population_density aged_65_older time IDDE IDECO IDEDU IDPOP IDAGE IDTIME

outreg2 using Moderate1,word
